# The Effects of Comprehensive Warm-Up Programs on Proprioception, Static and Dynamic Balance on Male Soccer Players

**DOI:** 10.1371/journal.pone.0051568

**Published:** 2012-12-12

**Authors:** Abdolhamid Daneshjoo, Abdul Halim Mokhtar, Nader Rahnama, Ashril Yusof

**Affiliations:** 1 Sports Centre, University of Malaya, Kuala Lumpur, Malaysia; 2 Faculty of Medicine, University of Malaya, Kuala Lumpur, Malaysia; 3 Faculty of Physical Education and Sport Science, University of Isfahan, Isfahan, Iran; Universidad Europea de Madrid, Spain

## Abstract

**Purpose:**

The study investigated the effects of FIFA 11+ and HarmoKnee, both being popular warm-up programs, on proprioception, and on the static and dynamic balance of professional male soccer players.

**Methods:**

Under 21 year-old soccer players (n = 36) were divided randomly into 11+, HarmoKnee and control groups. The programs were performed for 2 months (24 sessions). Proprioception was measured bilaterally at 30°, 45° and 60° knee flexion using the Biodex Isokinetic Dynamometer. Static and dynamic balances were evaluated using the stork stand test and Star Excursion Balance Test (SEBT), respectively.

**Results:**

The proprioception error of dominant leg significantly decreased from pre- to post-test by 2.8% and 1.7% in the 11+ group at 45° and 60° knee flexion, compared to 3% and 2.1% in the HarmoKnee group. The largest joint positioning error was in the non-dominant leg at 30° knee flexion (mean error value = 5.047), (p<0.05). The static balance with the eyes opened increased in the 11+ by 10.9% and in the HarmoKnee by 6.1% (p<0.05). The static balance with eyes closed significantly increased in the 11+ by 12.4% and in the HarmoKnee by 17.6%. The results indicated that static balance was significantly higher in eyes opened compared to eyes closed (p = 0.000). Significant improvements in SEBT in the 11+ (12.4%) and HarmoKnee (17.6%) groups were also found.

**Conclusion:**

Both the 11+ and HarmoKnee programs were proven to be useful warm-up protocols in improving proprioception at 45° and 60° knee flexion as well as static and dynamic balance in professional male soccer players. Data from this research may be helpful in encouraging coaches or trainers to implement the two warm-up programs in their soccer teams.

## Introduction

Balance or postural control can be defined as the ability to maintain a base of support with minimal movement and as the ability to perform a task while maintaining a stable position. Balance is maintained through dynamic integration of internal and external forces and factors involving the environment [1]–[3]. The regulation of balance depends on the visual, vestibular, and proprioceptive stimuli [2]–[4].

Static balance may be assessed by having an individual maintain a motionless position while standing on one or both legs [5]. Whereas, dynamic balance can be assessed by controlling the centre of mass with one leg while the other leg is reaching for maximum distance. The dynamic balance test has a greater demand on the balance and neuromuscular-control systems [6], [7].

Knee joint proprioception, which is essential for sufficient movement and stability, can best be illustrated as the afferent information arising from proprioceptors positioned in the capsules, ligaments, and muscle spindles that contributes to joint stability, postural control, and motor control [8]–[10].Proprioception is a specialized variation of the sensory modality and encompasses the sensations of joint movement (kinaesthesia) and of joint position (joint position sense). Joint position sense pertains to the accuracy of position replication, and is an individual’s ability to reproduce a predetermined joint angle. Proprioception is an important factor for promoting functional stability in playing soccer [2], [11]. A decline in proprioceptive function is seen following injury e.g. in anterior cruciate ligament tear of the knee [12] and may predispose to recurring injury. Thorp et al. [13], demonstrated that soccer players with functional ankle instability and poor balance were at significantly increased risk of ankle sprain re-injury [13]. In a recent systematic review by Hubscher et al. [14], seven methodologically well-conducted studies were pool-analysed on the effectiveness of proprioceptive training in reducing the incidence of injuries, including acute knee injuries and ankle sprains, finding significant risk reduction in both. [14]. They recommended that with this evidence, future research should focus on comparative trials to identify the most appropriate and effective training components for preventing injuries in specific sports and populations [14]. Thus, assessment of proprioceptive function is valuable in identifying proprioceptive deficits and the subsequent planning of appropriate preventative and rehabilitative programs [9], [15].

Several studies have considered the effects of different training programs on static balance [16], [17], dynamic balance [18], [19] and proprioception [2], [8], [20] in soccer players. The 11+ and the HarmoKnee are two new specific soccer warm-up programs. FIFA Medical and Research Centre (F-MARC) developed “The 11+” warm-up program for soccer players. The 11+ program which is an advanced version of the “11”, includes 27 exercises with a set of balance exercises [21]. Recently Kiani et al. [22] devised the HarmoKnee warm-up program comprising five parts- warm-up, muscle activation, balance, strength, and core stability [22]. Both programs can be performed and integrated into regular soccer practice sessions and require no additional equipment. One important element incorporated into both programs is the balance exercise. The balance exercise provides additional challenge to maintaining core stability and appropriate alignment [21].

**Table 1:** Stature characteristics of the subjects (values are mean±SD).

Groups	11+ (n = 12)	HarmoKnee (n = 12)	Control (n = 12)
Age (y)	19.2±0.9	17.7±0.4	19.7±1.6
Height(m)	1.81±5.1	1.79±6.4	1.83±4.6
Mass(kg)	71.7±4.6	71±7.6	76.4±5.8

It is known that training can optimally develop balance abilities in young people. Effgen [23] reported that 10-days of balance exercise programs improved the length of time that 45 deaf children could stand on one leg at a time [23]. Paterno et al. [24] concluded that three 90-minute neuromuscular training sessions per week for 6 weeks improved single-limb stability in young female athletes [24]. McLeod et al. (2009) found that 6-weeks of neuromuscular training can increase the dynamic balance (SEBT) and proprioceptive capabilities of young basketball players [19]. Six-week intervention programs consisting of balance and strength elements can increase the dynamic postural control (SEBT reach distance) of healthy young males and females [18]. Break dance exercise (twice a week) for two months can improve static balance in 9 year-old soccer players [25].

**Figure 1:**
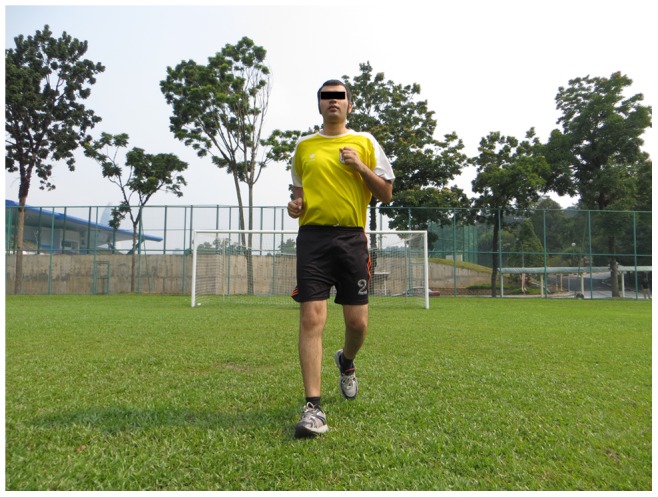
Running straight ahead.

**Figure 2:**
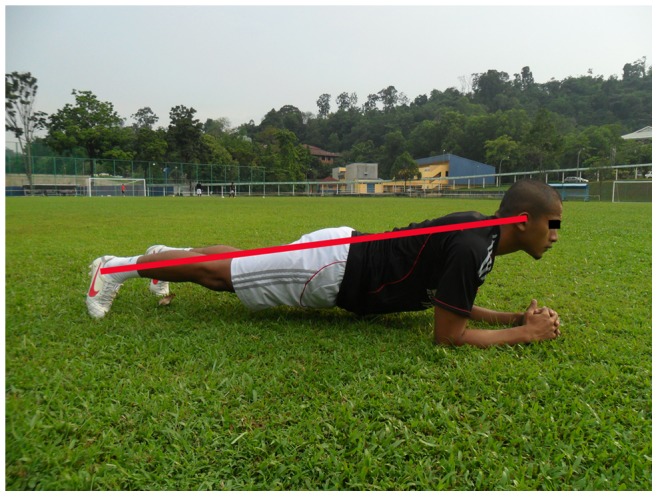
The bench static.

**Figure 3:**
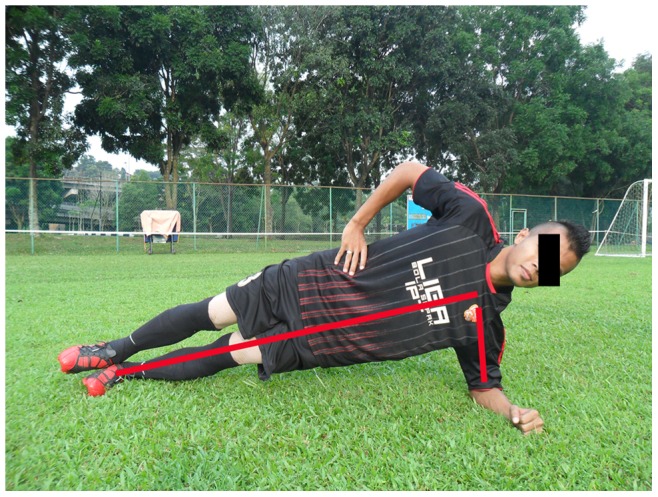
Sideways bench.

**Figure 4:**
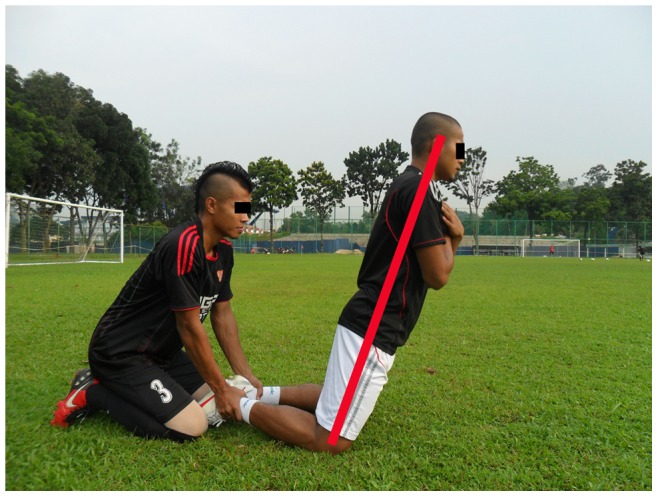
Nordic hamstring.

**Figure 5:**
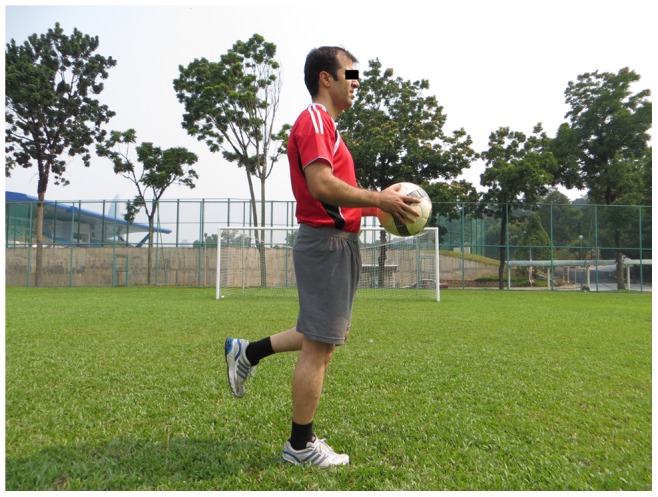
Single-leg stance, hold the ball.

**Figure 6:**
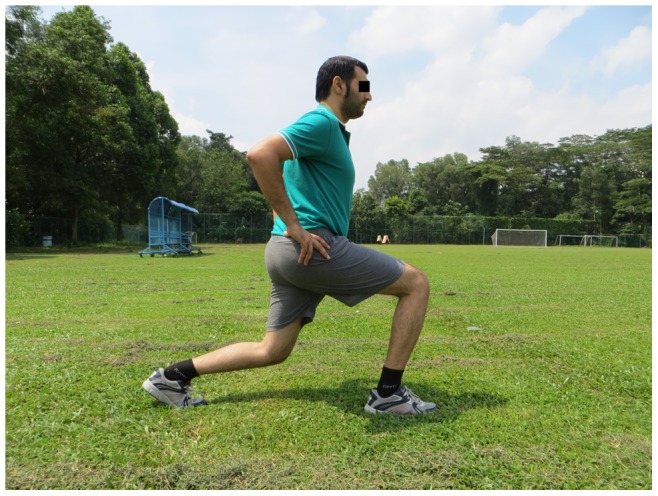
Walking lunges.

**Figure 7:**
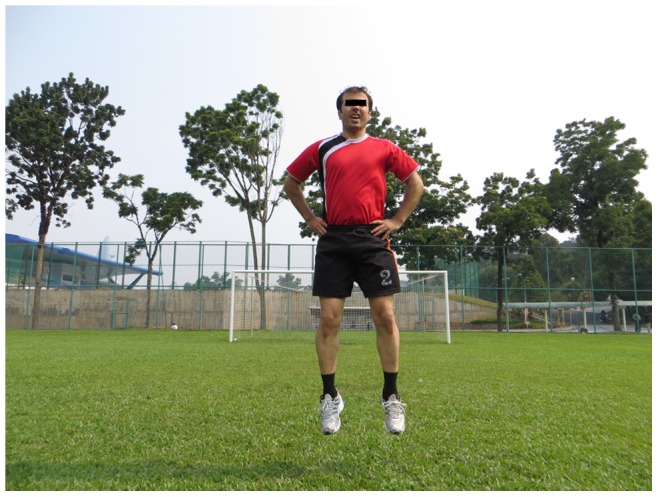
Vertical jumps.

**Figure 8:**
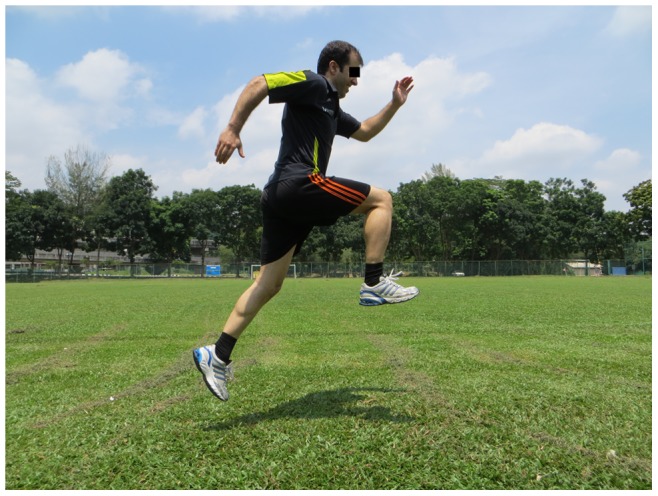
Bounding.

**Table 2:** The FIFA 11+. Exercises, duration and intensities of the structured warm-up program used (F-MARC).

Exercise	Duration	Figures
**Part 1: Running**	**8 minutes**	
Straight ahead, hip out, hip in, circling partner, shoulder contact, quick forward & backwards (6 running items, each item 2 sets)		*Figure 1*
**Part 2: strength, plyometric and balance**	**10 minutes**	
**The bench:** Static, alternate legs and one leg lift and hold (3 items, each item 3 sets)		*Figure 2*
**Sideways bench:** Static, raise & lower hip, with leg lift (3 items, 3 sets on each side)		*Figure 3*
**Hamstring:** Beginner (3–5 repetition, 1 set), intermediate (7–10 repetition, 1 set), advanced (12–15 repetition, 1 set). (3 items)		*Figure 4*
**Single-leg stance:** Hold the ball, throw the ball to a partner, test your partner (3 items, each item 2 sets)		*Figure 5*
**Squats:** With toe raise, walking lunges, one-leg squats (3 items, each item 2 sets)		*Figure 6*
**Jumping:** Vertical jumps, lateral jumps, box jumps (3 items, each item 2 sets)		*Figure 7*
**Part 3: running exercise**	**2 minutes**	
Across the pitch, bounding, plant & cut (3 items, each item 2 sets)		*Figure 8*

To our knowledge, there is no research that has investigated the effect of these two popular comprehensive warm-up programs on static and dynamic balance among professional soccer players. Moreover, with attention to the population of male soccer players which constitutes 239 million of all (270 million) registered soccer players [26], the aim of this study was to evaluate the effects of the two warm-up programs on proprioception, and on static and dynamic balance on male soccer players.

## Methods

### Ethics Statement

All the participants were informed orally about the procedures they would undergo and their written consents were taken. Moreover, we also obtained written informed consents from coaches, as caretakers, on behalf of the minor involved in this study. The study was approved by the ethical committee of the Institute of Research Management and Monitoring, University of Malaya and the Sports Centre Research Committee.

**Figure 9:**
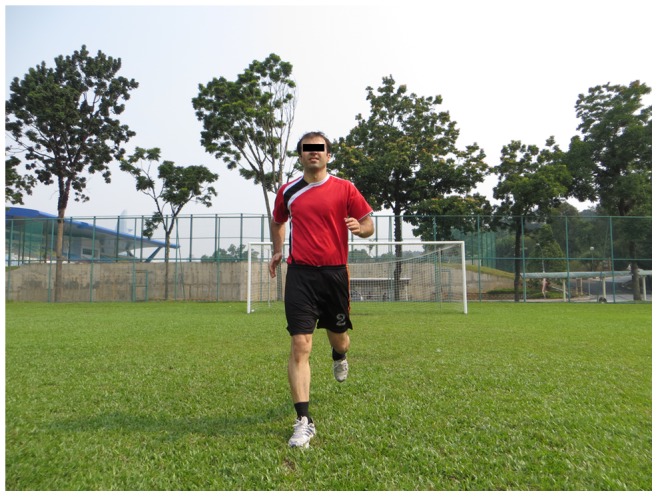
Jogging.

**Figure 10:**
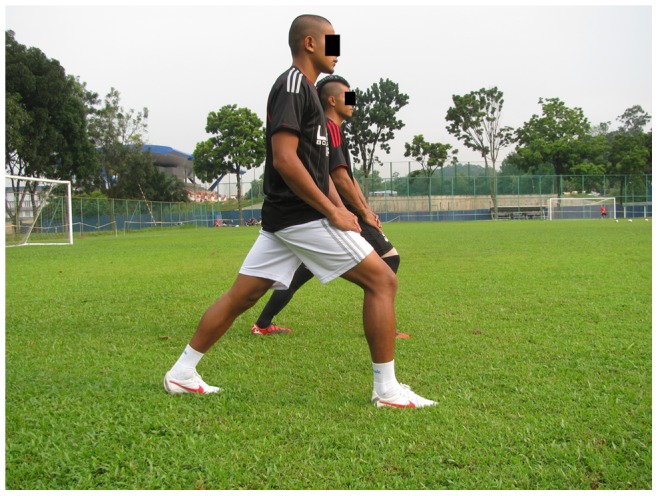
Activation of calf muscles.

**Figure 11:**
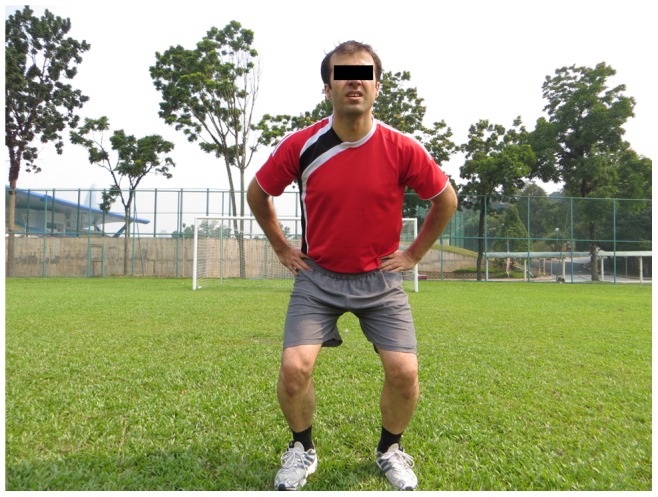
Double leg jump.

**Figure 12:**
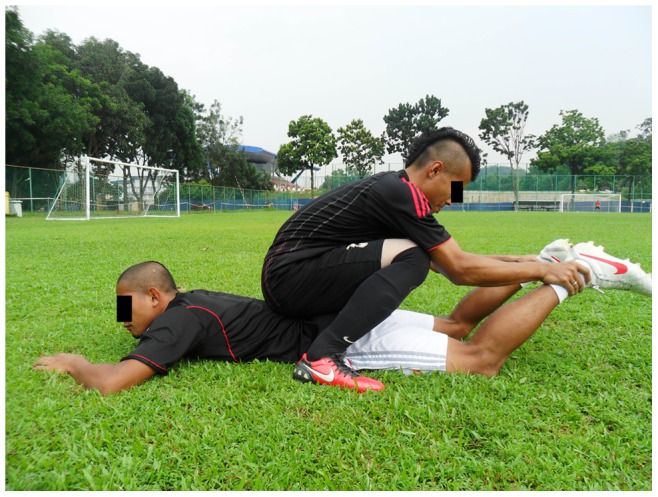
Hamstring curl (in pairs).

**Figure 13:**
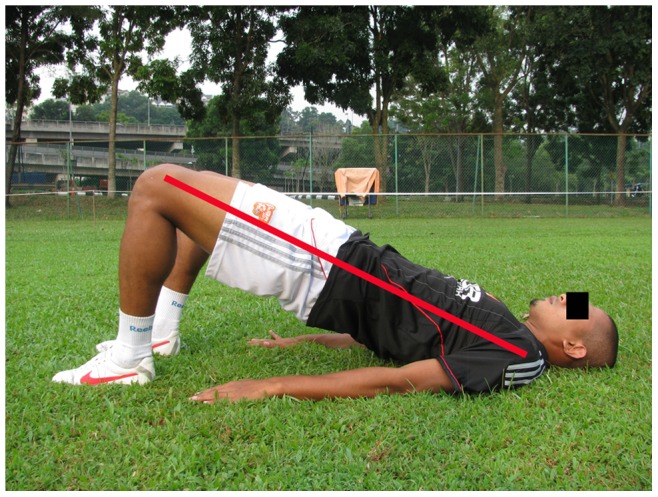
Bridging.

**Table 3:** The HarmoKnee training program, Exercises and duration of the structured warm-up program used.

Exercise	Duration	
**Warm-up**	**≥10 min**	
Jogging (≥4–6 min), Backward jogging on the toes (Approximately 1 min), High-knee skipping (Approximately 30 s), Defensive pressure technique (Approximately 30 s), One and one (≥2 min)		*Figure 9*
**Muscle activation**	**Approximately 2 min**	
Activation of calf muscles, quadriceps muscles, hamstring muscles, hip flexor muscles, groin muscles, hip and lower back muscles (6 item, each item 4 s for each leg/side)		*Figure 10*
**Balance**	**Approximately 2 min**	
Forward and backward double leg jumps, Lateral single leg jumps, Forward and backward single leg jumps, Double leg jump with or without ball (optional), (4 items, each item approximately 30 s)		*Figure 11*
**Strength**	**Approximately 4 min**	
Walking lunges in place, Hamstring curl (in pairs), Single-knee squat with toe raises (3 item, each item Approximately 1 min)		*Figure 12*
**Core stability**	**Approximately 3 min**	
Sit-ups, Plank on elbows and toes, Bridging (3 items, each item approximately 1 min)		*Figure 13*

### Participants

Thirty-six male young (between 17 and 20 years-old) who were professional soccer players (n = 36) with at least five years’ experience of playing soccer at professional level and training regularly without history of major lower limb injury or disease, participated in this study (Table 1). Players involved in martial arts or dancing, which may influence balance abilities, were excluded from this study. Three top professional teams (selected according to match outcome in the previous premier league) were chosen for this study.

**Figure 14:**
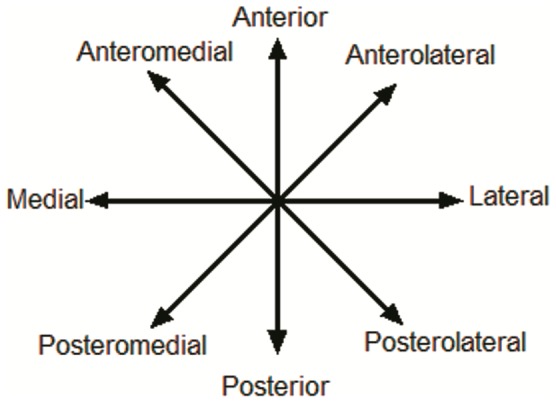
Star Excursion Balance Test (SEBT).

**Figure 15:**
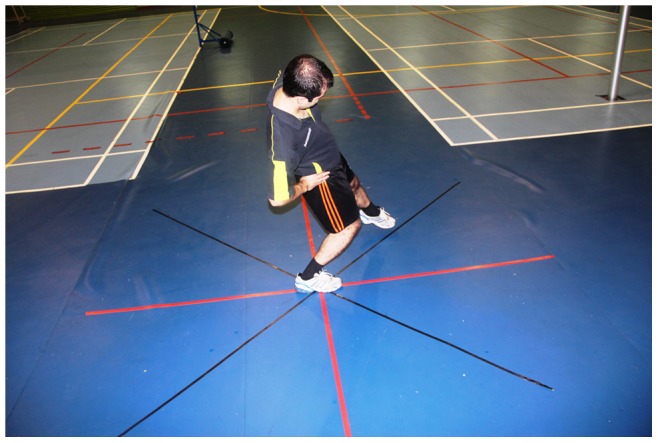
Anterior medial direction of the SEBT with a right stance leg.

### Procedure

At the mid-season of 2011, coaches and team managers from three professional teams were invited to a four-hour instruction course aiming to introduce the warm-up intervention programs. Three under 21years of age (U21) teams volunteered to participate in this study. The players from one team were randomly selected and assigned to one of the intervention programs. Each team had about 30 professional players, and from these, 12 matched players were randomly picked to participate in the study.

**Table 4:** Proprioception of groups in the dominant and non-dominant legs (values are mean ± SD) from pre-test to post-test.

	Dominant			Non-dominant		
Proprioception	pre	post	95%CI	pre	post	95% CI
**The 11+**						
30°	5.3±2.3	3.5±2.1	−4.01 to 0.5	5.6±3.5	4.3±2.8	−3.8 to 1.2
45°	6.0±2.6	3.2±1.8	−4.4 to −1.2**	5.8±2.8	4.2±3.4	−5.03 to 1.9
60°	4.7±2.1	3.1±1.4	−3.1 to −0.24*	5.1±3.1	3.5±2	−3.9 to 0.9
**HarmoKnee**						
30°	5.6±3.7	4.7±4	−4.9 to 3.0	6.6±3.6	4.1±2.3	−5.9 to 0.9
45°	6.0±2.9	2.9±1.5	−5.2 to −0.9**	3.5±2.6	3.2±1.7	−2.1 to 1.6
60°	4.7±2	2.6±2.4	−4.2 to −0.03*	3.6±1.4	3.3±2	−2.2 to 1.7
**Control**						
30°	5.4±3.5	3.3±1.8	−4.7 to 0.5	5.2±2.2	4.3±2.4	−3.2 to 1.4
45°	4.7±2.3	3.9±2	−2.7 to 1.1	3.6±2.4	3.4±2.3	−2.1 to 1.9
60°	5.7±3.2	4.1±2.6	−4.6 to 1.5	6.4±6.4	5.7±7.1	−2.4 to 1.1

Legend: pre = pre-test; post = post-test, ° = degree; CI = confidence interval; * p<0.05; **p<0.01.

Before starting the intervention programs, all players attended a workshop to discuss the prescribed training in detail and all players received video instructions and illustrations on the intervention programs. The players were also instructed on how to perform the exercises correctly. All training sessions were supervised by one of the researchers to ensure compliance with the programs. Before starting the proprioception test, verbal encouragements were given to help the subjects to concentrate on the quality of their movements. The exercise prevention programs started on 15^th^ April 2011 and concluded on 15^th^ June 2011 (24 sessions).

**Figure 16:**
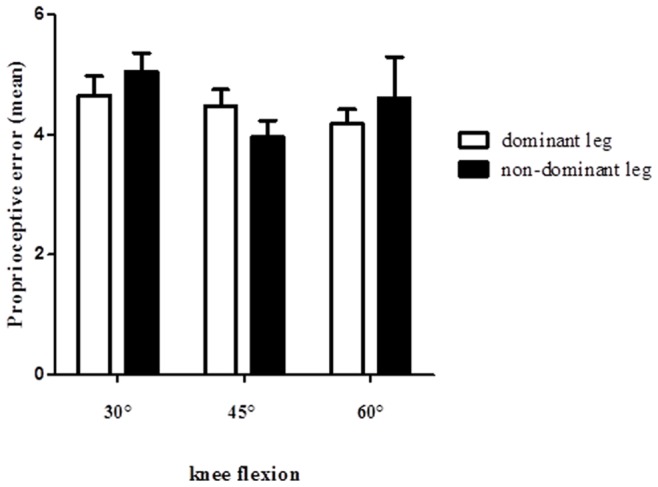
Proprioceptive errors in dominant and non-dominant legs.

### The Prevention Programs

#### The 11+ program

The 11+ consisted of three parts, beginning with running exercises (part I). Moving on to six exercises having three levels of increasing difficulty that developed strength, balance, muscle control and core stability (part II). Ending with advanced running exercises (part III). The different levels of difficulty improved the program’s efficiency and enabled coaches and players to individually adapt to the program. The 11+ took approximately 20–25 minutes to complete and replaced the usual warm-up before training. All exercises (27 exercises) focused on core stability, neuromuscular control, eccentric hamstring strength and agility (Table 2), (Figures 1–8). These exercises were performed three times per week.

**Table 5:** Static and dynamic balance in the groups (values are mean±SD) from pre-test to post-test.

	pre-test	post-test	95%CI
**11+ group**			
Static balance (EO) (s)	42.3±3.3	53.2±5.6	0.4 to 21.5*
Static balance (EC) (s)	17.9±2.6	30.3±5.4	1.7 to 23.2*
SEBT (cm)	97.2±9.4	103.9±5.6	2.7 to 10.7**
**HarmoKnee**			
Static balance (EO) (s)	41±5.1	47.1±8.8	1.7 to 10.6**
Static balance (EC) (s)	16.4±7.2	34±6.4	3.1 to 32.1*
SEBT (cm)	103.6±6.2	109.2±5.5	1.6 to 9.7**
**Control**			
Static balance (EO) (s)	40.9±4.8	44.2±3.5	2.3 to 8.9
Static balance (EC) (s)	17.7±4.3	19.9±4.2	6.02 to 10.5
SEBT (cm)	97.7±5.9	98.4±3.9	2.1 to 3.5

Legend: pre = pre-test; post = post-test, CI = confidence interval; EO = eyes opened; EC = eyes closed; s = second; cm = centimetre; * p<0.05; **p<0.01.

#### The HarmoKnee program

The HarmoKnee prevention program was designed by Kiani et al. [22]. The HarmoKnee is a multifaceted, soccer specific warm-up prevention program that combines education, proper motion patterns, strength and balance, and which aims at achieving an improved movement pattern and reducing knee injuries. The training protocol consists of five parts, warm up, muscle activation, balance, strength, and core stability. The parts of the program can be combined and performed during regular soccer training season (Table 3), (Figures 9–13). The total program duration was 20 to 25 minutes. Similar to the 11+, the HarmoKnee was also performed three times per week.

#### Control group

For comparison, the control group was asked to continue on with their regular training and warm-up without any restrictions.

### Proprioception Test

A Biodex Isokinetic Dynamometer (Biodex 3, 20 Ramsay Rode, Shirley, New York) was used to assess the proprioception of the subjects. All tests were carried out between 8 am and 11 am. Before each testing session, the gravity correction method of the dynamometer was chosen in accordance with the manufacturer’s recommendations. The subjects performed a general cardiovascular warm-up for at least 5 minutes on a Monark cycle ergometer at a moderate pace (50–100 W) followed by a 10-minute dynamic stretching concentrating on the lower body [27], [28].

Each subject was seated on the chair and assumed his most comfortable position. The subject was secured with snug straps across the shoulder, chest and hip. The cuff of the dynamometer’s lever arm was attached to proximal malleoli of the ankle. Dynamometer orientation was fixed at 90° and tilted at 0°, while the seat orientation was fixed at 90° and the seatback tilted at 70–85°. The rotational axis of the knee joint was aligned with the dynamometer rotational axis. All the seating positions of the subjects were recorded carefully and repeated during post-test. The order of testing was randomized for the dominant and non-dominant legs during pre-test. For assessment of proprioception, the tests were performed twice. The pre-test was conducted one week prior to the first day of training and the post-test was recorded eight weeks after the pre-test (three days after the final training session). All tests were conducted in the same order for each player, at pre- and post-tests [28], [29]. The testing was performed by the same member of the research team. The tester was blinded to each subject's intervention group.

The knee joint proprioception was investigated at target angles 30°, 45° and 60° in active mode using the Biodex 3systems. To memorize the target angles, participants’ legs were passively moved to the target angles (30°, 45° and 60° of knee flexion) [30], [31], their knees were held at the target position for 5 seconds, and then returned to the starting position (90°). The order of the 3 selected target angles was randomized to avoid any learning effect. Then, the participants were asked to extend their knees toward the previously selected target angle. The subjects were instructed to press the stop button when the memorized target angles were reproduced. The tests were performed three times for each target angle. Each test trial was conducted three times after a 30-second rest in a quiet place [32]. During the experiments, subjects were blindfolded and wore headphones to rule out visual and audible clues [32], [33]. The mean joint positioning error of the three measurement degrees of error from the target position were recorded for analysis [34]. The lower mean error value indicated the better knee proprioception.

### Dynamic Balance Test

Dynamic postural control was evaluated using the Star Excursion Balance Test (SEBT) [4], [19] that is carried out on a grid of eight lines (Figure 14). The foot of the dominant leg (the leg that was used to kick a ball defined as the dominant leg) [35], was positioned in the center of the grid, so that the foot was bisected equally in the anteroposterior and medial-lateral planes. SEBT consists of 8 reaching directions: anterior, anteromedial, medial, posteromedial, posterior, posterolateral, lateral and anterolateral (Figure 15). Subjects were requested to reach as far as possible along the designated line, lightly touching the line on the ground with the most distal part of the reaching foot, and returning the reaching leg back to double-leg stance, while maintaining a single-leg stance with the other leg in the center of the grid. They performed the test in a clockwise or counterclockwise manner, depending whether the dominant leg was the right or the left, respectively. Subjects were instructed to keep their hands on their iliac crests and to keep the heel of their stance leg on the ground at all times. Before the test trials during each test session, subjects performed six practice trials to provide a warm-up and overcome any learning effect. After the six practice trials, subjects were given a 5-minute rest period before performing the test trials. They were given as much time as they needed between trials, so that fatigue could be avoided. During the test trials, the reach distances were recorded with a mark on the tape line at the point of maximal reach and measured from the center of the grid. The average of the three reaches was normalized by dividing by the previously measured leg length to standardize the maximum reach distance ((excursion distance/leg length)×100 = % maximum reach distance). The higher excursion distance reflected the greater dynamic balance. Leg length was defined as the length measured from an anterior superior iliac spine (ASIS) to the medial malleolus tibia. A trial was discarded and repeated if the investigator noted the subject used the reaching leg for a substantial amount of support at any time, removed the supporting foot from the center of the grid, or was unable to maintain balance on the support leg throughout the trial [3], [5].

### Static Balance Test

Postural static balance was evaluated using the stork stand balance test [36]–[38]. In this test, the subject stood on his dominant leg. The participants were instructed to lift and hold the contralateral leg against the medial side of the knee of the stance leg while keeping his hands on the iliac crests. The trial ended when the heel of the involved leg touched the floor, the hands came off of the hips, or the opposite foot was removed from the stance leg. This test was conducted with eyes opened and eyes closed. The players performed three attempts and the best time was recorded for analysis [36]–[38].

### Statistical Analysis

To compare the proprioception within groups (pre-test, post-test), between groups (11+, HarmoKnee, control groups) and target angles (30°,45°,60°) the 2×3×3 (time vs group vs angle) mixed ANOVA was used. In case of statistical significance, the post-hoc Bonferroni test was also conducted. A mixed ANOVA (3×2×2) was applied for static balance to examine the possible interaction between group, time and eye (eyes opened and closed). A mixed ANOVA (3×2) was applied for SEBT dynamic balance to examine the possible interaction between groups and within group (pre- and post–tests) intervention phases. Furthermore, the Kolmogorov-Smirnov was employed for assessing normality of the distribution of scores. The Levene's test was employed for assessing homogeneity of variance among groups. A significant level was accepted at the 95% confidence level for all statistical parameters (p<0.05).

## Results

The Levene's test showed that the assumption of equal variances had not been violated for all variables (p>0.05). Furthermore, the Kolmogorov-Smirnov showed normality of the distribution of scores (p>0.05).

### Proprioception between Pre- and Post-tests

The means of proprioception error in pre-test and post-test of the groups are presented in Table 4. The mixed ANOVA indicated significant main effect between time in the dominant leg (F_1,33_ = 23.96, p = 0.000) and non-dominant legs (F_1,33_ = 4.57, p = 0.040). In the dominant leg the results did not show significant interaction between time with group (F_2,33_ = 0.244, p = 0.785), and time with angle (F_2,66_ = 0.246, p = 0.783). In the non-dominant leg results did not show significant interaction between time with group (F_2,33_ = 0.317, p = 0.730), and time with angle (F_2,66_ = 0.775, p = 0.465). The Bonferroni post-hoc test indicated significant differences between times at 45° knee flexion in the 11+ group (p = 0.003) and the HarmoKnee group (p = 0.009). Also The Bonferroni post-hoc test showed significant differences between times at 60° knee flexion in the 11+ group (p = 0.026) and the HarmoKnee group (p = 0.047). The Bonferroni post-hoc test did not showed any differences in 30° knee flexion (p>0.05). In the dominant leg of the 11+ group, results indicated a decreasing of mean error (p<0.05) by 2.8% and1.7% at 45°, 60° respectively. The results showed significant decrease in the dominant leg of the HarmoKnee group 3%, and 2.1% at 45°, 60° respectively. Moreover the post-hoc test did not show significant differences (p>0.05) in the non-dominant leg (pre- to post-tests).

### Knee Angle Comparison (30°, 45°and 60°)

The mixed ANOVA did not show significant difference between the target angles in the dominant leg (F_1,33_ = 0.706, p = 0.501). The mean of proprioceptive error value in the dominant leg were 4.651, 4.467 and 4.172 at 30°, 45° and 60°, respectively. But results indicated significant differences between angles in the non-dominant leg (F_1,33_ = 4.71, p = 0.016). The largest joint positioning error in the non-dominant leg was at 30° knee flexion (mean at 30°, 45° and 60°; 5.047, 3.956 and 4.613, respectively) (Figure 16).

### Comparison of Proprioception between Groups

The mixed ANOVA did not show significant difference between the intervention groups and control group in the dominant (F_2,33_ = 0.174, p = 0.841) and non-dominant legs (F_2,33_ = 0.560, p = 0.577).

### Comparison of Static Balance between Groups

The means of static balance in pre- and post-test of the groups are presented in Table 5. There is a significant main effect in static balance with the eyes open between time (pre- and post-tests), (F_2,33_ = 12.36, p = 0.001). However, no significant main effect were found between groups (F_2,33_ = 1.035, p = 0.366). The results did not show significant interaction between time with group (F_2,33_ = 3.101, p = 0.058). The Bonferroni post-hoc test indicated significant increases in static balance with eyes opened in the 11+ (p = 0.043) by 10.9% and the HarmoKnee (p = 0.011) by 6.1%. Significant differences were found in static balance with eyes closed between pre- and post-test (F_2,33_ = 12.80, p = 0.001). The Bonferroni post-hoc results indicated significant increases in the 11+ (p = 0.027) by 12.4% and the HarmoKnee (p = 0.022) by 17.6%. The results indicated significant differences between static balance with the eyes opened and closed (F_1,33_ = 74.420, p = 0.000).

### Comparison of SEBT between Groups

The means of dynamic balance in pre-test and post-test of the groups are presented in Table 5. There is a significant main effect between the time in SEBT (F_2,33_ = 20.42, p = 0.000). The results showed significant interaction between time with group (F_2,33_ = 3.767, p = 0.034). The Bonferroni post-hoc showed that SEBT in the 11+ (p = 0.004) and HarmoKnee (p = 0.011) groups were significantly increased by 6.7% and 5.6%, respectively. Significant main effect were found between groups during both pre- and post-tests (F_2,33_ = 6.77, p = 0.003). The Bonferroni post-hoc test indicated significant differences between the HarmoKnee (p = 0.003) and the control groups.

## Discussion

The present intervention study reports the effects of FIFA’s 11+ and the HarmoKnee injury prevention training programs on proprioception, and on static and dynamic balance of professional male young soccer players. Measurement of postural control is an important tool used to evaluate an athletic populations’ level of neuromuscular function in order to prevent injury [5]. The results revealed significant differences in mean proprioceptive errors between time in the dominant and non-dominant legs in the 11+ and HarmoKnee programs. However, only the dominant leg results indicated significant decreases from pre-test to post-test by 2.8% and 1.7% in the 11+ group, while 3% and 2.1% in the HarmoKnee group at 45°and 60°, respectively. The 11+ and HarmoKnee programs are multifaceted soccer specific prevention programs that include balance, core stability and neuromuscular control components [21], [22]. Balance training may lead to task-specific neural adaptations which may suppress spinal reflex excitability, such as the muscle stretch reflex during postural tasks, leading to less destabilizing movements and improved balance [39], [40]. These adaptations may cause influences on motor responses and may explain the improvement in knee proprioception from balance component [39], [40]. One cause for enhancement in proprioception following neuromuscular training, is that these exercises improve the concentration paid to proprioceptive cues by the brain, first at the conscious level early in exercise and then finally at the autonomous level [41]. Subasi et al. [2] studied the effects of warm-up programs on knee proprioception at 15°, 30°, and 60° knee flexion, and on balance in healthy young people. They reported that warm-up programs have positive effects on knee proprioception and balance [2]. During warm-up, the muscle tissues assess proper viscoelastic properties and body temperature, and together with improved oxygenation, will lead to enhance mechanoreceptor sensitivity [2], [42], [43]. These alterations enhance the functioning of mechanoreceptors and kinesthetic sensitivity [2]. It can be concluded that the 11+ and HarmoKnee programs have potential to improve knee proprioception. In addition, further modification of both programs may be required to fully improve knee proprioception. We suggest more training elements aimed to improve proprioception and balance should be added in both programs. Core stabilization training program consists of howling abdomen exercises, bridging, bird dogs [44] and also walking and running in backward directions [45] are recommended for improving static and dynamic balance.

The largest joint positioning error was found in the non-dominant leg at 30° knee flexion (5.047 mean error value at 30° angle versus 3.956 and 4.613 at 45°, 60°, respectively). Tsiganos et al. [34] investigated knee joint positioning sense using an isokinetic dynamometer at 30°, 45° and 70° knee flexion. They reported that the mean error value at 30° knee flexion was statistically greater than the mean error value at 70° knee flexion [34].The starting position to reproduce the leg extension target angles was at 90° knee flexion. Hence, the participants might have moved their knees to reproduce 30° knee flexion more than for 45° and 60°. This might cause more proprioceptive error in 30° knee flexion than other target angles. A second potential explanation might be related to the state of muscle spindles. The muscle spindles are the major contributors to proprioception [46]. As a muscle stretches and contracts, the muscle spindle is stimulated differently, and the magnitude of this stimulation also differs. To complicate this, the knee angle at which the muscle spindle is stimulated also has some bearing on the amount of stimulation on the muscle spindle [46], [47]. Relatively higher contraction is needed to produce the 30° target angle compared to 45° and 60° knee flexion. Residual cross bridges between the myofilaments actin and myosin may reciprocally increase muscle spindle activity in the antagonist muscle which may compromise proprioception [39].

The results showed significant differences in static balance (eyes opened and eyes closed) after 8-week intervention in the 11+ and HarmoKnee groups. Judge et al [48] reported that multifaceted exercise, which included strength and postural control balance components, improved 17% mean displacement of the centre of pressure in static balance in 21 older women [48]. The balance training led to improvement in neuromuscular facilitation, which enhanced static balance by suppressing the spinal reflex excitability (such as the muscle stretch reflexes), and improved agonist-antagonist muscle co-contraction [39], [49].

There were significant differences between static balance with eyes opened and eyes closed where static balance was better in the former. Giagazoglou et al. [17] compared static balance between blind and sighted women. They reported that vision plays a superior role than the coding and processing of other sensory information. The studies have reported that visual input affected neural control of body sway and that postural sway increases in the absence of vision [17], [50], [51].

Our findings revealed significant differences in SEBT from pre- to post-tests in the 11+ (12.4%) and HarmoKnee (17.6%) groups. The FIFA 11+ and HarmoKnee programs are multifaceted soccer specific programs that include Nordic hamstring, single leg stance, and squat and plyometric trainings [21], [22]. Leavey et al. [18] reported that 6 week combined exercise including balance and strength element programs can improve dynamic postural control (evaluated by SEBT) in healthy male and female [18]. McKeon et al. [52] reported that four weeks of balance training significantly improved dynamic postural control as assessed using the SEBT. They pointed out that this enhancement may be referred to the decrease in constraints placed on the sensorimotor system as a result of balance training [52]. Interestingly, when compared between groups, our findings in SEBT showed significant differences between the HarmoKnee and control group. The HarmoKnee program has more impact than the 11+ program on increasing SEBT in young male professional soccer players. Further research that investigates which components in the HarmoKnee program contributed to its significance is underway.

### Conclusion

We found that both warm up programs improved proprioception in the dominant leg at 45° and 60° knee flexion. The largest joint positioning error in non-dominant leg was at 30° knee flexion. The static balance (eyes opened and closed) in both groups definitely increased. Dynamic balance as assessed by SEBT also showed improvement in both groups, with the HarmoKnee group showing significant difference when compared to the control group. The two warm up programs have been shown to objectively improve proprioception and balance which in turn may improve performance and prevent injuries particularly lower limb injuries.
